# Practice of ventilation in critically ill pediatric patients: protocol for an international, long–term, observational study, and results of the pilot feasibility study

**DOI:** 10.62675/2965-2774.20250398

**Published:** 2025-05-13

**Authors:** Relin van Vliet, Jonathan Willem Jochem Melger, Frederique Paulus, Reinout Alexander Bem, Robert Gorge Theodoor Blokpoel, Marcus Josephus Schultz, David Michael Paul van Meenen, Martin Christiaan Jacques Kneyber

**Affiliations:** 1 Amsterdam University Medical Centers Department of Intensive Care Amsterdam The Netherlands Department of Intensive Care, Amsterdam University Medical Centers - Amsterdam, The Netherlands.; 2 University Medical Center Groningen Beatrix Children's Hospital Division of Paediatric Critical Care Medicine Groningen The Netherlands Department of Paediatrics, Division of Paediatric Critical Care Medicine, Beatrix Children's Hospital, University Medical Center Groningen - Groningen, The Netherlands.; 3 Amsterdam University Medical Centers Department of Anesthesiology Amsterdam The Netherlands Department of Anesthesiology, Amsterdam University Medical Centers - Amsterdam, The Netherlands.

**Keywords:** Critical illness, Intensive care units, pediatric, Noninvasive ventilation, Respiration, artificial, Ventilator weaning, Ventilators, mechanical, Pandemics, Child

## Abstract

**Objective:**

This manuscript describes the protocol of an investigator-initiated, international, multicenter, long-term, prospective observational study named PRactice of VENTilation in PEDiatric Patients (PRoVENT-PED), designed to investigate the epidemiology, respiratory support practices and outcomes of critically ill pediatric patients.

**Design:**

Data will be collected biannually over 10 years during predefined 4-week intervals, with an additional optional period to accommodate data collection during an epidemic or pandemic. The specific focus of PRoVENT-PED will evolve as the study progresses, initially emphasizing collecting detailed ventilator data from invasively ventilated patients. In later phases, the focus will shift to noninvasive respiratory support and typical aspects of respiratory support, like patient-ventilator asynchronies, weaning practices, and rescue therapies, as extracorporeal support. PRoVENT-PED includes patients under 18 years of age, admitted to a participating intensive care unit, and receiving respiratory support. The endpoints vary with the focus in each phase but will always include a set of key settings and ventilation parameters and related outcomes. If applicable, potentially modifiable factors and associations with outcomes will be studied. The pilot feasibility study demonstrated that the electronic capturing system effectively collects all necessary data within a reasonable time limit, with little missing data.

**Conclusion:**

PRoVENT-PED is a 10-year, international, multicenter study focused on collecting data on respiratory support practices in critically ill pediatric patients. Its scope evolves from invasive to noninvasive ventilatory support, ultimately encompassing patient-ventilator asynchronies, weaning practices, and rescue therapies.

## INTRODUCTION

Critically ill patients often require respiratory support,^(1)^ frequently involving invasive or noninvasive ventilation. Numerous studies in adult patients have shown significant variation in respiratory support practices, which have evolved.^(2-5)^ These studies informed clinical trials that ultimately transformed care for critically ill adults, improving outcomes through evidence-based adjustments - for instance, in ventilator settings, noninvasive support strategies, and other respiratory support practices, enhancing patient outcomes.^(6-14)^

The need for respiratory support is similarly prevalent among critically ill children. However, research in pediatric populations remains scarce, leading to, amongst others, a reliance on data from adult studies to guide pediatric respiratory support practices.^(15,16)^ This reliance may be misguided, as physiological differences, including lung and thorax compliances, susceptibility to ventilator-induced lung injury (VILI),^(17)^ and age-related differential responses to pulmonary injury^(18,19)^ and oxygen toxicity,^(20,21)^ suggest that adult findings may not translate directly to pediatric patients.

We designed a long-term, prospective observational study, named PRactice of VENTilation in PEDiatric Patients (PRoVENT-PED) focused on collecting comprehensive data on respiratory care practices in critically ill pediatric patients. The study employs an adaptive design, allowing its scope to evolve over the 10 years. Initially, it will prioritize collecting detailed ventilator data from invasively ventilated patients. In later phases, the scope will shift to various noninvasive respiratory support and more specific aspects of invasive ventilation. The feasibility of PRoVENT-PED was rigorously tested in two Dutch hospitals to confirm its suitability for broader implementation.

## METHODS

### Design and ethics

PRoVENT-PED is an investigator-initiated, long-term, prospective, international, multicenter study that will collect detailed respiratory support data biannually over 10 years during predefined 4-week intervals, with an optional additional period to accommodate data collection during epidemics or pandemics. International and regional networks of pediatric intensivists and respiratory therapists are responsible for recruiting participating centers. Study coordinators guide national and regional coordinators in acquiring ethical approval, and help in the administrative processes. Due to its observational nature, participation may not require individual patient consent in most nations or regions; however, this depends on national and regional legislation, and local policies of the participating centers. The study protocol was initially approved by the Institutional Review Board of the University Medical Center Groningen, the Netherlands (METc 2023/375). PRoVENT-PED is registered at Clinicaltrials.gov (study identifier NCT06220825). PRoVENT-PED complies with the International Conference on Harmonization-Good Clinical Practice (ICH-GCP).

### Adaptive study

**Figure 1:**
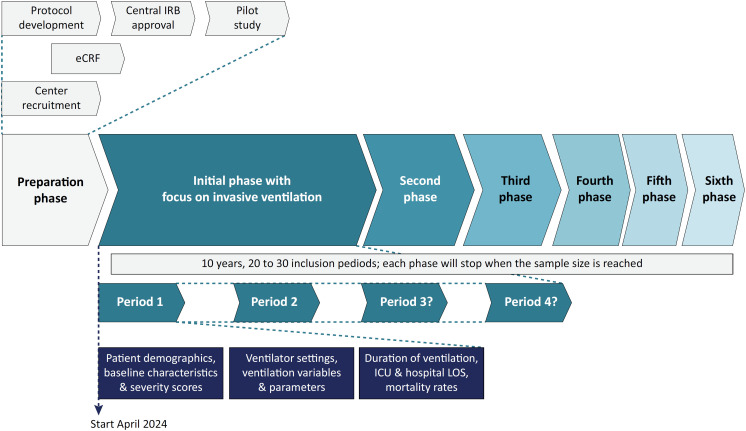
The schematic presentation of PRoVENT-PED highlights its adaptive design.

PRoVENT-PED employs an adaptive design, allowing the study's scope to evolve over the years the study is conducted (Figure 1). Initially, the study will prioritize the collection of comprehensive and detailed ventilator data from patients who are receiving invasive ventilation, as outlined in the following sections. The scope of each successive phase will be thoroughly outlined in a study amendment and documented on Clinicaltrials.gov to ensure transparency and provide ongoing updates on the study's evolving objectives.

Each phase, comprising one or more periods, focuses on critical issues and emerging developments. The duration of each phase is determined by calculating the sample size based on the study question for that phase.

IRB - Institutional Review Board; eCRF - electronic Case Report Form; PARDS - pediatric acute respiratory distress syndrome.

### Patients

Overall, patients are eligible for participation in PRoVENT-PED if: (1) under 18 years of age; (2) admitted to the ICU of a participating hospital during the period of data collection; and (3) receiving respiratory support for at least 12 hours, which ranges from simple supplementary oxygen or high-flow nasal oxygen (HFNO) to noninvasive ventilation and invasive ventilation, with or without extracorporeal membrane oxygenation (ECMO). During each phase, the study's evolving focus will adapt the inclusion criteria. In the initial phase, with the emphasis on invasive ventilation, receiving invasive ventilation will serve as an additional inclusion criterion. In the subsequent phases, the focus will shift to noninvasive respiratory support, accompanied by revised inclusion criteria that reflect this change in emphasis.

Preterm infants, i.e., patients with a postconceptional age corrected for gestational age below 40 weeks, are excluded from participation. Naturally, with each shift in focus, the exclusion criteria will also change during each phase. For example, if the study's emphasis transitions to noninvasive ventilatory support, patients who are receiving only invasive ventilation will be excluded from participation.

### Data collection

All data to be collected are part of standard clinical care. Throughout each phase of the study, the data collected will be tailored to align with the evolving focus of the research, ensuring that it remains relevant and reflects the specific objectives of each stage. At admission, we will collect data on patient demographics - including age, gestational age, sex, height and weight, comorbidities, and baseline characteristics - including reasons for intensive care unit (ICU) admission, and the start of respiratory support, presence of the pediatric acute respiratory distress syndrome (PARDS), and disease severity scores. For 4 days, beginning with the day of start of respiratory support, daily data will be collected at a time in the morning - including data on respiratory support and the respiratory status. Follow-up will be until day 28 to collect outcome data, including the date of stop of respiratory support, date of ICU discharge, and life status at ICU discharge or at day 28, whichever comes first.

Investigators will transcribe all collected data into a web-based electronic Case Report Form (eCRF) (Castor Electronic Data Capture, Amsterdam, the Netherlands).^(22)^ The eCRF complies with all applicable laws and regulations. Transcription will only take place in a secure environment. Directly identifying personal data will be separated from the research data and replaced by an assigned code. The directly identifying data will only be used to contact the patients and will only be available to the local investigators. Access to the assigned codes linked with personal data will be password-protected, restricting access to only the data directly related to the local investigator.

### Study endpoints

The study endpoints vary based on the study's evolving scope. Since the scope of the initial phase of PRoVENT-PED is invasive ventilation, key endpoints include a set of ventilator settings and ventilation parameters, such as tidal volume (V_T_); maximum (Pmax), peak (Ppeak), plateau (Pplat) and mean airway pressures (Pmean); positive end-expiratory pressure (PEEP); fraction of inspired oxygen (FiO^2^); and respiratory rate (RR) at fixed time points over the first four days of invasive ventilation. Driving pressure (ΔP), respiratory system compliance (C_RS_), and measures of ventilation intensity are calculated daily from these data. Other endpoints include duration of ventilation in survivors and nonsurvivors, the number of days free from invasive ventilation and alive at day 28, length of stay in ICU and hospital, and mortality rates in ICU and hospital and at day 28.

### Definitions

Day 0 of invasive ventilation is defined as the day respiratory support is started in the participating ICU; in the initial phase, this is the day at which invasive ventilation is started. Day 1 is defined as the first full calendar day, with Day 2 and Day 3 representing the two consecutive mornings that follow. Pediatric acute respiratory distress syndrome is defined according to the 2023 Pediatric Acute Lung Injury Consensus Conference (PALICC-2) definition.^(23)^ Intensive care unit length of stay is defined as the time between ICU admission and discharge or death; duration of respiratory support, in the initial phase invasive ventilation, is defined as the time between initiation and successful liberation. Intensive care unit mortality is defined as any death until ICU discharge or day 28, whichever comes first.

### Calculations

Ventilator-free days at day 28 (VFD-28) is calculated as 28 minus the number of days a subject was successfully liberated from ventilation after initiation. If a patient dies within 28 days, VFD-28 will be recorded as zero. The V^T^ is normalized to actual body weight (ABW), and ΔP,^(8)^ C_RS_,^(24)^ mechanical power (MP)^(25)^ and mechanical energy (ME) of ventilation,^(26)^ are calculated using the following equations 1 to 9:


[Equation 1] $${\text{V}}_{\text{T}}\text{normalized to ABW}={\text{V}}_{\text{T}}/\text{ABW}$$

[Equation 2] ΔP=Pplat−PEEP in volume-controlled ventilation (VCV)

[Equation 3] ΔP=Pmax−PEEP in pressure-controlled ventilation(PCV)

[Equation 4] $${\text{C}}_{\text{RS}}={\text{V}}_{\text{T}}/(\text{Pplat}-\text{PEEP})(\text{with VCV})$$

[Equation 5] $${\text{C}}_{\text{RS}}={\text{V}}_{\text{T}}/(\text{Pmax}-\text{PEEP})\text{(with PCV)}$$

[Equation 6] $$\text{MP}=0.098^{*}\;{\text{V}}_{\text{T}}*\text{RR}*(\text{Ppeak}-0.5^{*}\Delta \text{P})(\text{with VCV})$$

[Equation 7] $$\text{MP}=0.098^{*}\;{\text{V}}_{\text{T}}*\text{RR}*(\mathrm{Pmax}-0.5^{*}\Delta \text{P})(\text{with PCV})$$

[Equation 8] $$\text{ME}=0.098^{*}\;{\text{V}}_{\text{T}}/{\text{kg}}^{*}(\text{Ppeak}-0.5^{*}\Delta \text{P})$$

[Equation 9] $$\text{ME}=0.098^{*}{\text{V}}_{\text{T}}/{\text{kg}}^{*}(\mathrm{Pmax}-0.5^{*}\Delta \text{P})$$

### Sample size calculation

The sample size will vary across each phase. In the initial phase, which focuses on invasive ventilation, the sample size calculation is based on identifying potentially modifiable factors independently associated with ICU mortality. We plan to include up to ten modifiable factors in the model to achieve this. Using logistic regression and the rule of thumb of ten events per factor, we estimated a need for one hundred events’, that is ICU deaths. Based on a conservative ICU mortality rate estimate of 3%,^(1,27)^ 3,000 patients will be required. However, depending on the actual mortality rate observed with each 4-week interval, the sample size may be adjusted accordingly. For subsequent phases, in which the study's focus and analysis plan will shift, sample sizes will be recalculated and reported in the study amendment and on Clinicaltrials.gov.

### Randomization

To prevent larger centers from disproportionately influencing the findings, we will implement randomization in centers expected to enroll more than one hundred patients during a given period, based on the focus of each phase.

### Statistical analysis

Descriptive statistics will be used to describe patient demographics and baseline characteristics, types of respiratory support used, device settings, and respiratory variables and parameters, including measures of oxygenation and decarboxylation. Continuous variables will be expressed in medians and interquartile ranges, and categorical variables will be expressed in frequencies and proportions. The Kruskal-Wallis test will be used to compare continuous variables, and the Chi-squared test will be used for categorical variables.

Respiratory support settings and respiratory variables and parameters at successive days will be reported in detail for the first days of respiratory care and compared between different age groups, i.e., neonates (< 1 month), infants (1 month to < 12 months), toddlers (12 months to < 3 years), preschoolers (3 years to < 6 years), school-age children (6 years to < 12 years), and adolescents (12 years to 18 years). Additionally, respiratory support settings, respiratory variables, and ventilation parameters will be compared between patients with and without PARDS and across other patient groups, including patients receiving respiratory support due to a pulmonary disease *versus* non-pulmonary conditions and patients receiving ECMO.

Key respiratory support settings, respiratory variables, and parameters will be visualized using cumulative distribution plots for the first days and line graphs over successive days. Inpatient trends over time are assessed with mixed-effect linear models with patients treated as a random effect to account for clustering and repeated measurements, and with day, different patient group and their interaction as fixed effects. P values from this analysis represent the overall difference among different patient groups over time, and p values from interaction represent the difference in trend over time among the different patient groups.

Time-to-event outcomes, like liberation from the ventilator, discharge and death, will be presented in Kaplan-Meier curves. Cox proportional hazard modelling or competing risk analyses will be used to study associations with continuous clinical outcomes, presented as hazard ratios (HR) and 95% confidence intervals (95%CIs). Logistic regression will be used to study the association between key ventilator settings and ventilation parameters and binary clinical outcomes, and will be presented as odds ratios (OR) and 95%CIs.

A robust locally weighted scatter plot smoothing (LOWESS) method will be used to investigate the association between ICU mortality and key ventilatory variables and parameters, including V^T^, Ppeak, PEEP, FiO^2^, RR, PaO^2^/FiO^2^, ΔP, MP and ME. The smoothing curves will be generated with a bandwidth of 0.66, a polynomial regression with 1 degree of freedom, and a tricubic weighting function so that observations furthest from the point of interest are assigned the least weight.

In a univariate analysis, the impact of the single key ventilatory variables on mortality will be assessed by estimating the relative risk of all-cause 28-day ICU mortality. Multivariate logistic regression will be used for the adjusted analysis, adjusting for unequal distribution of effect modifiers. We will adjust for baseline covariates selected according to clinical relevance, including age, sex, weight, comorbidities, and severity of illness. Results will be shown as ORs with 95%CI. Multiple imputation will be performed to account for missing values.

Data will be analyzed with R version 4.2.2 (R Core Team, 2022, Vienna, Austria). Statistical uncertainty will be expressed by 95%CIs. A p value < 0.05 is considered statistically significant.

### Study organization

The executive committee, composed of a study coordinator, two principal investigators, and four additional investigators, oversaw the protocol's design, the development and testing of the eCRF, the organization of meetings, and the provision of support to study coordinators. The advisory committee, comprising experts in pediatric respiratory support, refined and finalized the protocol and will offer intellectual guidance throughout the study. National coordinators within participating ICUs are responsible for securing local ethical approval, establishing data transfer agreements, and assisting local investigators. National coordinators and local investigators handle patient enrolling patients and data collection. National coordinators and local investigators are collectively responsible for ensuring the quality and security of the data collected.

### Patient and public involvement

The study does not involve patients or the public at any stage of its design, conduct, or analysis.

### Dissemination

The finding from each PRoVENT-PED phase will be reported per the Strengthening the Reporting of Observational Studies in Epidemiology (STROBE) guidelines.^(28)^ The study outcomes will be published in peer-reviewed journals. After the primary results have been disseminated, the pooled dataset will be accessible for secondary analysis upon request, subject to the Steering Committee's assessment and approval of the scientific merit and validity of the proposed analysis.

### Feasibility study

#### Design, oversight and ethics

To evaluate the feasibility of PRoVENT-PED and the eCRF used for data collection in its initial phase, we conducted a pilot study to assess the time required to gather all necessary data for each included patient and to evaluate data completeness. In January and February 2023, and again in July and August 2023, we screened patients for eligibility in two Dutch pediatric ICUs: The University Medical Center Groningen (UMCG) and the Netherlands’ Amsterdam University Medical Centers (AUMC). The Institutional Review Board's approval covered this pilot for the entire study, and the need for individual patient informed consent was waived due to the study's observational nature.

#### Patients

We applied the inclusion and exclusion criteria as described above for the initial phase of the study, i.e., patients were eligible for participation in this pilot study if: (1) aged ≤ 18 years; (2) admitted to the pediatric ICU of a participating hospital; (3) for invasive ventilation for at least 12 hours.

#### Data collected

Data collection followed the plan as projected for the initial phase. Additionally, the time required for data entry was recorded for each patient.

## RESULTS

Of 398 patients screened, 125 were eligible for this pilot study, 91 from the UMCG and 34 from the AUMC (Figure 1S - Supplementary Material). The main reason for exclusion was not receiving invasive ventilatory ventilation. Patients were predominantly male, and most patients were neonates or infants (Table 1). The most common comorbidities were pulmonary diseases, syndromes, and genetic abnormalities. Most patients were admitted to the ICU and intubated due to pulmonary conditions, and 11% met PARDS criteria. All-cause 28-day ICU mortality was 8%, the median duration of ventilation was 4 [2 - 10] days, and the median length of ICU stay was 5 [3 - 11] days.

**Table 1:** Patient demographics, baseline characteristics and outcomes

Variables		
Patient demographics		
	Age (months)	8 [2 - 72]
	Sex (male)	80 (64)
	Body weight (kg)	8 [5 - 22]
	Body height (cm)	74 [55 - 120]
	Premature birth	14 (11)
Disease severity score		
	PIM III	1 [0 - 2]
Medical history		
	Pulmonary	26 (22)
	Cardiac	23 (18)
	Neuromuscular	5 (4)
	Syndrome or genetic abnormalities	32 (26)
	Oncologic	1 (1)
	Organ transplant	8 (7)
	Chronic ventilation	2 (2)
	Mental retardation	6 (5)
Reason for ICU admission		
	Respiratory	70 (57)
	Cardiac	8 (7)
	Neuromuscular	4 (3)
	Postoperative	32 (26)
	Home ventilation	1 (2)
	Renal failure	3 (2)
	Trauma	6 (5)
	Intoxication	1 (1)
Main reason for invasive ventilation		
	Respiratory	65 (52)
	Circulatory	13 (10)
	Neurological	9 (7)
	Metabolic	1 (1)
	Postoperative weaning	33 (26)
	Trauma	4 (3)
Barotrauma during admission		
	Pneumothorax	3 (3)
	Subcutaneous emphysema	1 (1)
	Pneumomediastinum	1 (1)
Tracheostomy		3 (2)
Discharged		106 (85)
Destination of discharge		
	ICU of another hospital	5 (4)
	Ward	91 (72)
	Other healthcare facility	6 (5)
	Home	4 (3)
Duration of ventilation (days)		4 [2 - 10]
Length of ICU stay (days)		5 [3 - 11]
28-day mortality		10 (8)

PIM III - Pediatric Index of Mortality III; ICU - intensive care unit. Data presented as median with interquartile range [25^th^ - 75th quartile] or n (%).

Median time to collect all data per patient was 20 [15 - 25] minutes (Table 1S - Supplementary Material), the amount of missing data was low (Table 2S - Supplementary Material) and considered acceptable, and local investigators expressed no difficulties using Castor EDC as eCRF for data entry.

**Figure 2:**
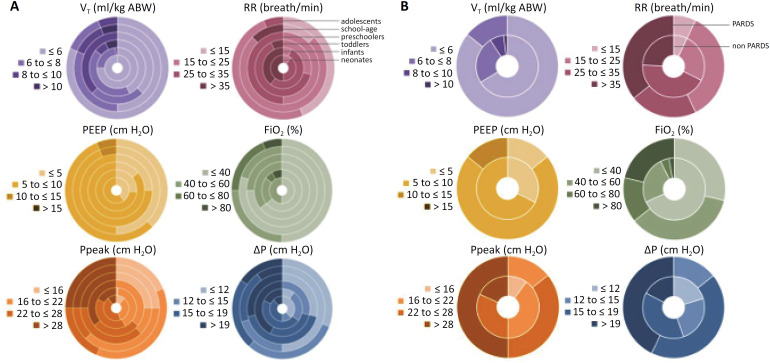
Ring graphs of key ventilator settings and ventilation variables and parameters at day 0, (A) categorized by age groups and (B) the presence or absence of pediatric acute respiratory distress syndrome.

The most used ventilation mode was PCV (92%) (Table 2). Most patients received V_T_ of less than 8mL/kg, with younger children receiving lower median V_T_ than older patients (Figure 2). Younger patients received lower median PEEP than older patients. Median FiO_2_ and median RR were higher, and median ΔP was lower in younger children. PARDS patients received lower V_T_, higher median PEEP and FiO_2_, and lower median ΔP than non-PARDS patients.

**Table 2:** Ventilator settings, ventilation variables and parameters, and arterial blood gas analysis at day 0

		All n = 125	Neonates n = 10	Infants n = 61	Toddlers n = 14	Preschoolers n = 8	School-aged n = 16	Adolescents n = 16
Ventilation mode								
	Volume controlled ventilation	9 (7)	0 (0)	0 (0)	0 (0)	1 (13)	5 (31)	3 (19)
	Pressure controlled ventilation	115 (92)	10 (100)	60 (98)	14 (100)	7 (88)	11 (68)	13 (81)
	Volume support	1 (1)	0 (0)	1 (2)	0 (0)	0 (0)	0 (0)	0 (0)
Ventilator settings								
	V_T_ (mL/kg ABW)	6 [5 - 7]	6 [5 - 6]	6 [5 - 7]	7 [4 - 8]	9 [7 - 9]	7 [6 - 8]	6 [5 - 7]
	PEEP (cmH_2_O)	6 [5 - 8]	7 [5 - 8]	6 [5 - 8]	7 [6 - 8]	7 [6 - 8]	6 [5 - 8]	7 [5 - 9]
	RR (breath/min)	30 [25 - 36]	36 [35 - 38]	35 [30 - 40]	29 [26 - 33]	26 [22 - 30]	20 [20 - 25]	19 [15 - 24]
	FiO_2_ (%)	40 [30 - 50]	45 [36 - 58]	40 [30 - 50]	40 [40 - 45]	38 [29 - 45]	30 [30 - 40]	45 [39 - 63]
Ventilation variables and parameters								
	Ppeak (cmH_2_O)	23 [20 - 27]	23 [20 - 26]	24 [21 - 27]	26 [22 - 30]	24 [18 - 26]	20 [17 - 26]	21 [19 - 28]
	Pmean (cmH_2_O)	12 [10 - 14]	10 [10 - 13]	12 [11 - 14]	13 [11 - 16]	13 [10 - 15]	10 [9 - 13]	11 [8 - 13]
	etCO_2_ (kPa)	6 [5 - 7]	5 [3 - 6]	6 [6 - 7]	6 [5 - 7]	5 [5 - 5]	5 [4 - 6]	5 [5 - 6]
	C_RS_	3 [2 - 7]	1 [1 - 2]	2 [1 - 3]	4 [3 - 5]	6 [5 - 10]	15 [11 - 18]	23 [20 - 26]
	ΔP (cmH_2_O)	16 [14 - 19]	17 [12 - 18]	17 [15 - 19]	18 [15 - 22]	17 [13 - 21]	13 [10 - 18]	14 [10 - 19]
	MP (J/minute)	2 [1 - 5]	1 [1 - 1]	2 [1 - 2]	3 [3 - 5]	5 [3 - 5]	5 [4 - 8]	8 [6 - 10]
	ME (mJ/kg)	9 [7 - 11]	10 [6 - 10]	8 [7 - 10]	11 [7 - 14]	11 [10 - 14]	10 [8 - 12]	9 [7 - 10]
Arterial/capillary blood gas analysis								
	pHa	7.4 [7.3 - 7.4]	7.4 [7.3 - 7.4]	7.3 [7.3 - 7.4]	7.3 [7.3 - 7.4]	7.4 [7.3 - 7.4]	7.4 [7.3 - 7.4]	7.4 [7.4 - 7.4]
	PaO_2_ (kPa)	6.7 [6.3 - 6.7]	5.2 [5.2 - 5.3]	6.7 [6.0 - 6.7]	6.7 [6.7 - 6.7]	6.7 [6.7 - 6.7]	6.7 [6.7 - 6.8]	6.7 [6.7 - 6.7]
	PaCO_2_ (kPa)	5.8 [5.2 - 6.6]	5.8 [5.4 - 6.3]	6.6 [5.6 - 6.7]	6.1 [5.8 - 6.3]	4.5 [4.2 - 5.2]	5.1 [4.7 - 5.7]	5.2 [5.0 - 5.7]

V_T_ - tidal volume; PEEP - positive end-expiratory pressure; RR - respiratory rate; FiO_2_ - fraction of inspired oxygen; Ppeak - peak pressure; Pmean - mean airway pressure; etCO_2_ - end-tidal carbon dioxide; C_RS_ - respiratory system compliance; ΔP - driving pressure; MP - mechanical power; ME - mechanical energy; pHa - arterial pH; PaO_2_ - partial arterial oxygen pressure; PaCO_2_ - partial arterial pressure of carbon dioxide. Data presented as n (%) or median with interquartile range [25^th^ - 75th quartile].

V_T_ - tidal volume; RR - respiratory rate; PEEP - positive end-expiratory pressure; FiO_2_ - oxygen fraction in inspired air; Ppeak - peak pressure; ΔP - driving pressure.

## DISCUSSION

This manuscript outlines the study protocol for PRoVENT-PED and reports the findings from its pilot feasibility study. PRoVENT-PED aims to enhance understanding of respiratory support practices in critically ill pediatric patients, encompassing invasive ventilation during the study's initial phase, transitioning to noninvasive respiratory support, and addressing key elements of respiratory and rescue therapies in subsequent phases. The pilot study demonstrated the feasibility of the electronic data capture system, enabling efficient data collection with minimal missing data.

Research into ventilatory support practices in critically ill children remains limited and outdated. Earlier studies identified substantial variability in key ventilation settings, but these findings are over a decade old and may not reflect current practice.^(29-41)^ Additionally, poor adherence to lung-protective strategies in PARDS has been demonstrated, likely reflecting the limited availability of robust evidence.^(42)^. Advances in lung-protective strategies for invasive ventilation and the increasing adoption of noninvasive ventilation and HFNO are likely to have influenced modern approaches.^(38,43-46)^ In adult populations, studies such as Large Observational Study to UNderstand the Global Impact of Severe Acute Respiratory FailurE (LUNG SAFE),^(3)^ PRactice of VENTilation in Critically Ill Patients without ARDS at Onset of Ventilation (PRoVENT)^(4)^ and PRactice of VENTilation in Middle-Income Countries (PRoVENT-iMiC),^(2)^ have highlighted significant differences in ventilation practices between patient groups and geo-economic regions, underscoring the impact of patient demographics and baseline characteristics, as well as local practices and resource availability on care. It is reasonable to expect similar variability in pediatric populations, necessitating contemporary data to inform future trials and guide implementing optimal respiratory support strategies for children.

The proposed study offers several strengths. PRoVENT-PED's adaptive design allows for a flexible focus on evolving clinical priorities, enabling the investigation of key topics such as invasive and noninvasive ventilation, weaning, patient-ventilator asynchronies, and extracorporeal support. Once the required sample size for one topic is reached, the study can seamlessly transition to the next, ensuring responsiveness to emerging challenges in pediatric intensive care. Its prospective design ensures more accurate data collection on exposures, confounders, and outcomes compared to retrospective studies, reducing the risk of bias and improving the reliability of the findings. The planned large sample size is expected to provide robust insights into associations between ventilation practice and clinical outcomes. By including diverse ICUs across resource-limited and resource-rich settings worldwide, the study captures variations in practice, patient populations and ventilation strategies, enabling exploration of geo-economic differences and their impact on outcome. Additionally, data collection across different seasons and patient groups will allow for comprehensive subgroup analyses, including patients with and without PARDS, various age categories, and diverse disease conditions.

A pilot study was conducted to test the feasibility of data collection and assess the presence of missing data. Most data were effectively captured. Notably, the absence of specific data does not necessarily indicate missingness but may reflect clinical practice. For instance, blood gas analyses might not have been performed if not clinically indicated, and such cases were not classified as missing data. In contrast, variables like patient height, which should have been recorded but were not, were treated as genuinely missing. This distinction was carefully accounted for in the analysis. Furthermore, the time required for data collection was considered sufficiently low, even when accounting for settings such as low- and middle-income countries (LMICs), where the time available for such tasks may be more limited.

The sample size for PRoVENT-PED will be adapted across the different phases of the study. In the initial phase, which focuses on invasive ventilation, the sample size calculation is designed to identify potentially modifiable factors independently associated with ICU mortality. However, the sample size may be adjusted based on the observed mortality rates during this inclusion period. In the pilot feasibility study, mortality was higher than predicted in the initial sample size calculation. If this finding is confirmed in the early periods of the study, it may allow for an earlier conclusion of the initial phase, as the required number of events would be reached sooner than anticipated. This adaptive design is a key strength of PRoVENT-PED, as it aligns recruitment efforts with observed clinical outcomes, minimizing unnecessary workload and ensuring efficient use of resources.

Ventilation practices in adult and pediatric ICUs continuously evolve, driven by emerging evidence and external factors such as pandemics. In adult patients, the adoption of HFNO has increased steadily, supported by positive findings across diverse clinical settings,^(47-53)^ including in the COVID-19 pandemic.^(14,54)^ Awake-prone positioning also became more prevalent^(55)^ while reliance on high PEEP strategies decreased.^(56-58)^ Similarly, pediatric ICUs have embraced noninvasive strategies with growing frequency,^(59-61)^ particularly HFNO, although evidence of benefit is lacking.^(43,45,46,62,63)^ The COVID-19 pandemic also influenced the epidemiology of pediatric respiratory infections, leading to fewer pediatric ICU admissions in 2020 and 2021, followed by a resurgence with greater severity and increased ventilatory support requirements.^(64-71)^ These evolving trends highlight the importance of ongoing research into respiratory support practices. A notable strength of PRoVENT-PED is its capacity to incorporate additional data collection during epidemics or pandemics, facilitating more timely and relevant insights.

PRoVENT-PED has several limitations. Selection bias may occur as centers interested in various types of ventilatory support are more likely to participate. We will invite ICUs through various organizations and networks to reduce this risk. Furthermore, since the study is unfunded, resource-rich ICUs may be overrepresented. We will attempt to mitigate this by offering full support to participating centers where feasible. Data collection could be challenging due to the involvement of ICUs across different countries, each with varying health record systems and spanning both resource-rich and resource-limited settings. To address this, we will streamline respiratory support data and limit follow-up to either ICU discharge with a maximum of 28 days, aiming to make the workload manageable for all participating centers.

Additionally, some patients may be lost to follow-up due to transfers from participating to non-participating centers. Finally, given its observational design, PRoVENT-PED can identify associations between practice and outcomes but cannot establish causality. Nevertheless, these associations will inform the design of future randomized clinical trials.

In conclusion, PRoVENT-PED is a well-scaled, long-term observational study with an adaptive, global design. This design enables the collection of extensive data across diverse patient populations and settings. This study will provide valuable insights that can enhance and guide respiratory care for critically ill pediatric patients.

### PRoVENT-PED investigators

Ragad Al Abdwani, Anis Siham Zainal Abidin, Lorena Acevedo, Alice Ackah, Anika Adam, Hasan Agin, Linda Agyekum, Ouissal Aissaoui, Nihal Akçay, Mehmet Alakaya, Ben Albert, Awokech Alemayehu, Abdullah Alghobaishi, Ahmad E. Alhaj, Hashim E. Alhashemi, Nasser Ambu Ali, Ammar al Alzadjali, Varun M. Angadi, Suresh Kumar Angurana, Ilse Appel, Eliisa Appelberg, John Adabie Appiah, Gazi Arslan, Didar Arslan, Ayse Asik, Gülhan Atakul, Gurkan Atay, Yvonne Avent, Marcella Aversa, Fevzi Aydoğdu, Tigist Bacha, Luis Pérez Baena, Patricia Barbosa de Carvalho, Angelina Beer, Reinout A. Bem, Merle Benstrom, Vanessa Bezerra Cerqueira Lobo, Richard Biedermann, Bronagh Blackwood, Robert Blokpoel, Laurence Boillat, Ezio Bonanomi, Matthew A. Borgman, Luise Brado, Sebastian Brenner, George Briassoulis, Nora Bruns, Juerg M. Burren, Leonardo Calil Vicente Franco de Souza, Felipe Caino, Cristina Camilo, Anna Camporesi, Fabio Joly Campos, Pedro Carballo Martin, Francesco Cardona, Nilton Yhuri Carreazo, Werther Brunow de Carvalho, Joanna Cena, Shubham Charaya, Markos Tomidis Chatzimanouil, Giovanna Chidini, Fabrizio Chiusolo, Elpida Chocliourou, Maria Cristina Mondardini, Eleni Christakou, Hans Cipowicz, Mustafa Colak, Lurisha Coopoosamy, Leonardo Costa, Leila Costa Volpon, Luís Fernando Andrade de Carvalho, Veronica Ferreira Cury, Evangelia Dardamani, Hasna Darouich, Linda G. Doedens, Swati Dublish, Els L.I.M. Duval, Muhterem Duyu, Shalom Dziekpor, Faruk Ekinci, Sanella Ellersich, Eziamaka Jennifer Enemuo, Marie-Sophie Esche, Jaime Fernández-Sarmiento, Thomas Ferry, Liza Fitria, Lucio Flavio Peixoto de Lima, Flávia Krepel Foronda, Ana M. Llorente de la Fuente, Angeliki Galani, Martina De Gaudenzi, Shira J. Gertz, Edwin Godson, Chen Yun Goh, Robert Graham, Kristina Grinko, Regina Grigolli Cesar, Shalu Gupta, Jaisson Gustavo da Fonseca, Hans Fuchs, Hye-ji Han, Young Joo Han, Walid M Hassan, Sabien G.J. Heisterkamp, Klara Horvath, Hoh Hong Huat, Stavroula Ilia, Mari-Liis Ilmoja, Asumthia S. Jeyapalan, Atul Jindal, Cintia Johnston, Pon Kah Min, Riste Kalamees, Murat Kangin, Utku Karaarslan, Mona Karimullah, Atsushi Kawaguchi, Yasser Kazzaz, Ayesha Bibi Khan, Majd Khiami, Elizabeth Y. Killien, Kyunghoon Kim, David Kiptum, Martin C.J. Kneyber, Çelebi Kocaoğlu, Alper Koker, Ali Korulmaz, Yoshida Kota, Kandamaran Krishnamurthy, Kevin van't Kruys, Charles Dekun Lai, Francesca Landolfo, Hans-Jörg Lang, Jan Hau Lee, Inés Leoz Gordillo, Rakesh Lodha, Jesus Lopez-Herce, Yolanda M. Lopez-Fernandez, Daniele M. de Luca, Maria Machaira, Fidaa Maghrabi, Ririe Fachrina Malisie, Ananya Manchikalapati, Alessandro Mantovani, Eleni Peristera Mantzafleri, Matthieu Maria, Eugene Martey, Alicia Rodríguez Martínez, Amelia Martínez-de-Azagra, Caroline Máximo Batista, Jennifer A. McArthur, David M.P. van Meenen, Claudia Mei Lan Jae, Jonathan W.J. Melger, Mehmet Emin Menentoglu, Harmen Mensink, Tuuli Metsvaht, Malgorzata Mikaszewska-Sokolewicz, Merve Misirlioglu, Shinya Miura, Marta R. Moniz, Amélia Moreira, Brenda M. Morrow, Jayashree Muralidharan, Susan Murphy, Karthi Nallasamy, Kuban D. Naidoo, Matteo Di Nardo, Michael A Nares, Ary Serpa Neto, Uchenna Nnajekwu, Nompi M. Nyindi, Çağlar Ödek, Samuel Nee-Okpey, Saad A Alotaibi, Farhana Al Othmani, Helgi Padari, Togara M. Pamacheche, Frederique Paulus, Phan Huu Phuc, Carla Regina Pinto, Luigi Pisani, Virginie Plante, Claire Procter, Hasim Qaitoon, Amine Rakrak, Habyna Ravichandrajah, Bhupinder Reel, Karl Reiter, Elizabeth Ricciardi, Thomas Riedel, Vikesh Rowjee, Astri Safitri, Robin T. Saggers, Manas Ranjan Sahoo, Shamiel Salie, Rujipat Samransamruajkit, Özlem Sarac Sandal, Paula Santos Herraiz, Miriam Santschi, Tommaso Sasso, Luregn Jan Schlapbach, János Schnur, Lukas Schroeder, Marcus J. Schultz, Alexandre Peixoto Serafim, Esra Sevketoglu, Shinya Shimoyama, Pablo Silvero, Melina Simon, Teresa Singer, Ekin Soydan, Kelly Straka, Paolo Taffache, Edwin Bin Tan, Xiaoliao Tang, Riccardo Tarquini, Rafael Teixeira Azevedo, Fadhilla Tekka, Leyla Telhan, David Tingay, Demet Tosun, Victoria Trautmann, Sirawut Trepatchayakorn, Javier Urbano, Pravin Vasanthan, Maria Vassilopoulou, Giacomo Veronese, David Arjona Villanueva, Relin van Vliet, Lee Siew Wah, Arnaud Wiedemann-Fode, Miranda Wiggelinkhuizen, Ariane Willems, Judith Wong Ju-Ming, Roelie M. Wösten-van Asperen, Long Xiang, Cheong Ching Yee, Abate Yeshidenber, Saptadi Yuliarto, Susan Zakariah, Giorgio Zampini, Wenlan Zhang and Elisa Zimmermann.
