# Supplementary material for: Practice of ventilation in critically ill pediatric patients: protocol for an international, long–term, observational study, and results of the pilot feasibility study
Source: Crit Care Sci. 2025 May 13;37:e20250398. doi: 10.62675/2965-2774.20250398 (PMC12094694; doi:10.62675/2965-2774.20250398)
Supplement: Supplementary file 1 [file 2965-2774-ccsci-37-e20250398-Suppl01.pdf]

Critical Care Science
https://doi.org/10.62675/2965-2774.20250398

SUPPLEMENTARY MATERIAL

Practice of ventilation in critically ill pediatric patients: protocol for an international, long–term, observational study, and results of the pilot feasibility study

Relin van Vliet[1], Jonathan Willem Jochem Melger[1], Frederique Paulus[1], Reinout Alexander Bem[1], Robert Gorge Theodoor Blokpoel[2], Marcus Josephus Schultz[1], David Michael Paul van Meenen[3], Martin Christiaan Jacques Kneyber[2], for the PRoVENT-PED investigators and the PROVE Network

Table 1S - Time used for data collection time in individual patients

Variables	
Total used time (min)	20 [15 - 24]
Inclusion (min)	1 [1 - 2]
Demographics (min)	1 [1- 2]
Medical history (min)	1 [1 - 1]
Disease severity score (min)	2 [2 - 2]
Daily visits (min)	12 [9 - 15]
Follow–up (min)	2 [1 - 2]

Times (min) represent the duration per patient. Data presented as median with interquartile range [25th - 75th quartile].

Table 2S - Missing data

	All n = 125	Neonates n = 10	Infants n = 61	Toddlers n = 14	Preschoolers n = 8	School–aged n = 16	Adolescents n = 16
Demographics							
Age (months)	0 (0)	0 (0)	0 (0)	0 (0)	0 (0)	0 (0)	0 (0)
Male	0 (0)	0 (0)	0 (0)	0 (0)	0 (0)	0 (0)	0 (0)
Weight (kg)	1 (1)	0 (0)	1 (2)	0 (0)	0 (0)	0 (0)	0 (0)
Height (cm)	19 (15)	4 (40)	11 (18)	0 (0)	2 (25)	2 (13)	0 (0)
History of premature birth	0 (0)	0 (0)	0 (0)	0 (0)	0 (0)	0 (0)	0 (0)
Medical history	0 (0)	0 (0)	0 (0)	0 (0)	0 (0)	0 (0)	0 (0)
Reason for ICU admission	1 (1)	0 (0)	0 (0)	0 (0)	0 (0)	1 (6)	0 (0)
Reason for intubation	0 (0)	0 (0)	0 (0)	0 (0)	0 (0)	0 (0)	0 (0)
Date of ICU admission	0 (0)	0 (0)	0 (0)	0 (0)	0 (0)	0 (0)	0 (0)
Disease severity score (PIM III)							
Systolic blood pressure (mmHg)	1 (1)	0 (0)	0 (0)	0 (0)	0 (0)	1 (6)	0 (0)
PaO_2 (kPa)	0 (0)	0 (0)	0 (0)	0 (0)	0 (0)	0 (0)	0 (0)
FiO_2 (%)	2 (2)	0 (0)	2 (3)	0 (0)	0 (0)	0 (0)	0 (0)

Continue...

This is an open access article under the CC BY license (https://creativecommons.org/licenses/by/4.0/).

...continuation

Pupil response	0 (0)	0 (0)	0 (0)	0 (0)	0 (0)	0 (0)	0 (0)
Base excess (mmol/L)	0 (0)	0 (0)	0 (0)	0 (0)	0 (0)	0 (0)	0 (0)
Reason of ICU admission	1 (1)	0 (0)	1 (7)	0 (0)	0 (0)	0 (0)	0 (0)
Risk (low/high/very high)	0 (0)	0 (0)	0 (0)	0 (0)	0 (0)	0 (0)	0 (0)
Ventilation variables							
Ventilation mode	0 (0)	0 (0)	0 (0)	0 (0)	0 (0)	0 (0)	0 (0)
V_T (mL)	0 (0)	0 (0)	0 (0)	0 (0)	0 (0)	0 (0)	0 (0)
PEEP (cm H_2O)	0 (0)	0 (0)	0 (0)	0 (0)	0 (0)	0 (0)	0 (0)
Ppeak (cm H_2O)	1 (1)	1 (10)	0 (0)	0 (0)	0 (0)	0 (0)	0 (0)
Pmean (cm H_2O)	10 (8)	0 (0)	5 (8)	0 (0)	1 (13)	2 (13)	2 (13)
Pplat (cm H_2O)	2 (2)	0 (0)	0 (0)	0 (0)	1 (0)	1 (0)	0 (0)
RR (breath/min)	0 (0)	0 (0)	0 (0)	0 (0)	0 (0)	0 (0)	0 (0)
Tinsp (seconds)	2 (2)	1 (10)	1 (2)	0 (0)	0 (0)	0 (0)	0 (0)
SpO_2 (%)	1 (1)	0 (0)	1 (2)	0 (0)	0 (0)	0 (0)	0 (0)
FiO_2 (%)	1 (1)	0 (0)	0 (0)	0 (0)	0 (0)	1 (6)	0 (0)
$etCO_2$ (kPa)	6 (5)	1 (10)	1 (2)	0 (0)	1 (13)	2 (13)	1 (6)
Blood gas results							
pHa	0 (0)	0 (0)	0 (0)	0 (0)	0 (0)	0 (0)	0 (0)
PaO_2 (kPa)	0 (0)	0 (0)	0 (0)	0 (0)	0 (0)	0 (0)	0 (0)
$PaCO_2$ (kPa)	0 (0)	0 (0)	0 (0)	0 (0)	0 (0)	0 (0)	0 (0)
Adjunctive therapies							
ECMO (VA/VV)	0 (0)	0 (0)	0 (0)	0 (0)	0 (0)	0 (0)	0 (0)
NMBA (continuous/bolus)	3 (2)	1 (10)	2 (3)	0 (0)	0 (0)	0 (0)	0 (0)
Prone positioning	1 (1)	0 (0)	0 (0)	0 (0)	0 (0)	0 (0)	1 (6)
Medication							
Sedatives (continuous/bolus)	2 (2)	0 (0)	2 (3)	0 (0)	0 (0)	0 (0)	0 (0)
Opioids (continuous/bolus)	2 (2)	0 (0)	2 (3)	0 (0)	0 (0)	0 (0)	0 (0)
Vasoactive (continuous/bolus)	3 (3)	1 (10)	2 (3)	0 (0)	0 (0)	0 (0)	0 (0)
PARDS criteria							
Perinatal lung disease (y/n)	0 (0)	0 (0)	0 (0)	0 (0)	0 (0)	0 (0)	0 (0)
Onset within 7 days (y/n)	0 (0)	0 (0)	0 (0)	0 (0)	0 (0)	0 (0)	0 (0)
Edema (y/n)	1 (1)	0 (0)	0 (0)	0 (0)	0 (0)	0 (0)	1 (6)
Chest imaging	0 (0)	0 (0)	0 (0)	0 (0)	0 (0)	0 (0)	0 (0)
Follow–up							
Life status	0 (0)	0 (0)	0 (0)	0 (0)	0 (0)	0 (0)	0 (0)
ICU discharge (y/n)	0 (0)	0 (0)	0 (0)	0 (0)	0 (0)	0 (0)	0 (0)
Destination of discharge	0 (0)	0 (0)	0 (0)	0 (0)	0 (0)	0 (0)	0 (0)
Date of discharge	0 (0)	0 (0)	0 (0)	0 (0)	0 (0)	0 (0)	0 (0)
Date of extubation	0 (0)	0 (0)	0 (0)	0 (0)	0 (0)	0 (0)	0 (0)
Tracheostomy (y/n)	1 (1)	0 (0)	0 (0)	0 (0)	1 (13)	0 (0)	0 (0)
Barotrauma	0 (0)	0 (0)	0 (0)	0 (0)	0 (0)	0 (0)	0 (0)

V_T - tidal volume; PEEP - positive end–expiratory pressure; Ppeak - peak pressure; Pmean - mean airway pressure; Pplat - plateau pressure; RR - respiratory rate; Tinsp - inspiration time; SpO_2 - peripheral oxygen saturation; FiO_2 - fraction of inspired oxygen; $etCO_2$ - end–tidal carbon dioxide; pHa - arterial pH; PaO_2 - partial arterial oxygen pressure; $PaCO_2$ -partial arterial pressure of carbon dioxide; ECMO - extracorporeal membrane oxygenation; NMBA - neuromuscular blocking agents - PARDS, and pediatric acute respiratory distress syndrome; ICU - intensive care unit. Data are n (%).

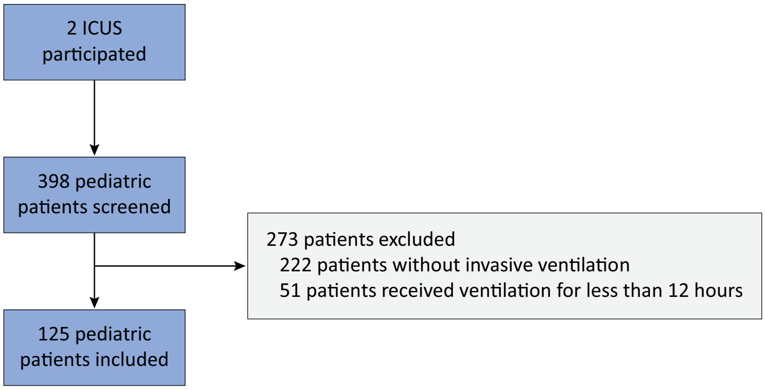

Figure 1S - CONSORT diagram.
Patient flow in the pilot study.
ICU - intensive care unit.
